# Microgravity impairs endocrine signaling and reproductive health of women. A narrative review

**DOI:** 10.3389/fphys.2025.1558711

**Published:** 2025-05-13

**Authors:** Michela Cutigni, Giorgia Cucina, Emanuele Galante, Matteo Cerri, Mariano Bizzarri

**Affiliations:** 1 Department of Experimental Medicine, Space Biomedicine Laboratory, University Sapienza, Rome, Italy; 2 Department of Biomedical and NeuroMotor Sciences, University of Bologna, Bologna, Italy

**Keywords:** weightlessness, steroidogenesis, aromatase, female fertility, ovarian function

## Abstract

During space exploration missions the organism is subjected to several challenges. Most of the studies have been performed on male health in space, leaving the focus on sex differences behind. With the development of new biological technologies, attention is now being paid more to how spaceflight conditions affect human reproductive health. In this review, the focus is on how weightlessness disrupts ovarian function and endocrine signaling by affecting the hypothalamic-pituitary-gonadal axis. Emerging evidence suggests that microgravity can impair estrogen production through the suppression of aromatase expression in granulosa cells. This condition leads to a hypo-estrogenic condition that harms the ovulation and the menstrual cycle. Likely, due to reduced estrogen availability, bone density, and cardiovascular health can consequently be severely involved. New studies focus on how space-related deregulation involving ovarian steroidogenesis look like the picture observed in the Polycystic Ovary Syndrome. These similarities open the perspective to counteract pharmacologically the observed abnormalities. However, our knowledge is severely constrained by the limited data available as well by the lack of proper experimental models of investigation. Indeed, much is required in order to acquire a full understanding of endocrine and functional changes occurring during microgravity exposure, including the joint effect of radiation and weightlessness that deserve to be thoroughly investigated to recognize the respective contribution of each one as well as the eventual synergies.

## Introduction: The importance of assessing the gonadal hormones in space

To undertake prolonged missions in deep space, and in the perspective for stable settlements on the Lunar or Mars surface, it is mandatory to assess how spaceflight conditions–radiation and microgravity exposure–interfere with gonadal functions and affect the reproductive capabilities. By February 2024, a total of 681 individuals have reached the altitude of space according to the USAF definition, with 610 achieving Earth orbit. Among them, 155 (22.7%) have been women.

The overall physiological and psychological adaptation to the spaceflight environment is similar for male and female astronauts. However, there are critical differences in physiological functioning related to sex (Ronca et al., 2014).

Noticeably, besides the well-known effects of radiation on the reproductive system (Ogilvy-Stuart and Shalet, 1993), microgravity can also modify the endocrine control of both male and female gonads (Mathyk B. et al., 2024).

Male and female reproductive systems are different and therefore deserve independent focus and research to understand the short and long-term impacts of the spaceflight environment. The female reproductive system presents a significant challenge for scientific investigation, as its endocrine regulation involves multiple interconnected levels—including the central nervous system, adrenal glands, and gonads—functioning according to a highly orchestrated timing.

Furthermore, throughout all its phases, female sexual and reproductive function is highly sensitive to a wide range of physical, chemical, and psychological stressors.

Therefore, understanding whether and how ovarian endocrine function is conserved in space conditions is mandatory to preserve the integrity of other systems, which are frequently hindered in space, especially due to weightlessness (Aydogan et al., 2024). Although pregnancy is contraindicated during spaceflight, in the forecast of permanent off-Earth human settlements, this aspect of human life in space should be considered. Indeed, female sex steroid hormones (estrogen, progesterone) have an impact on multiple body apparatuses, and they play a special role in modulating bone, muscle and cardiovascular functions, making a clear difference in respect to males. As we said above, certain conditions exhibit significant differences based on sex (Chromiak et al., 2003). However, no increase in gynecologic cancer risk has yet been revealed in the female astronaut population (Barr et al., 2007) but, as exploration missions will be outside of low Earth orbit and for increasingly long durations, concern remains regarding the effects related to prolonged exposure to both microgravity and even a low-dose rate of radiation accumulating over time (Drago-Ferrante et al., 2022).

Sadly, the distinctive features of the female endocrine system, which is more intricately connected to a periodic endocrine regulation of the organism, have been ignored for decades in biomedical space research, considering that the space community considered males a sufficient/appropriate proxy for all humans (Simon, 2005).

Lately, however, the overall impact of spaceflight weightlessness on reproductive function has been investigated in several studies and reviews (Gimunová et al., 2024), (Strock et al., 2023).

Recently, the ESA *SciSpacE* white papers set out the research needed to advance our knowledge in this field of space physiology and identified eight key knowledge gaps (ESA SciSpacE White Papers, 2021). Namely, the white paper recognizes that there is limited knowledge available on the systemic effects of spaceflight stressors upon the general endocrine control on both females and males. However, due to the small numbers of female astronauts who have undertaken long-duration spaceflight it was not possible to perform a direct assessment of the impact of space conditions upon female fertility. Therefore, it is required to deepen our understanding on how increased and prolonged spaceflight affect the functionality of reproductive organs, as well as the conceivable risk of cancer for offspring due to the exposure of germ cells to space stressors. To achieve such objectives, it is essential to identify appropriate experimental models both encompassing simulated weightlessness and experiments conducted aboard the International Space Station involving cells, tissues and organs as well as living animals. Reproductive capabilities of living organisms are challenged by both radiation exposure and microgravity, and these stressor factors can eventually synergize. We are aware of the impact of radiation on endocrine and reproductive apparatus, whereas microgravity consequences have been underestimated for a long time; given that, the mechanisms elicited by weightlessness are still a matter of research and deserve an appropriate investigation.

Therefore this review aims to examine the effects of microgravity on gonadal function and reproductive health, with a focus on female fertility. It wants to highlight the existing knowledge gaps, regarding endocrine regulation and long-term fertility risks in space, remarking the need for experimental models to ensure the feasibility of prolonged human space missions and future space settlements.

For the bibliography research, we began by defining the main research question to establish clear guidelines for selecting relevant sources. We then explored various academic databases, library catalogs, and reliable online repositories to gather books, journal articles, and other materials related to the topic. Once we identified the most suitable references, we organized them systematically and formatted them according to the appropriate citation style, ensuring accuracy and consistency throughout the bibliography.

## Assessing the reproductive function: main challenges

**FIGURE 1:**
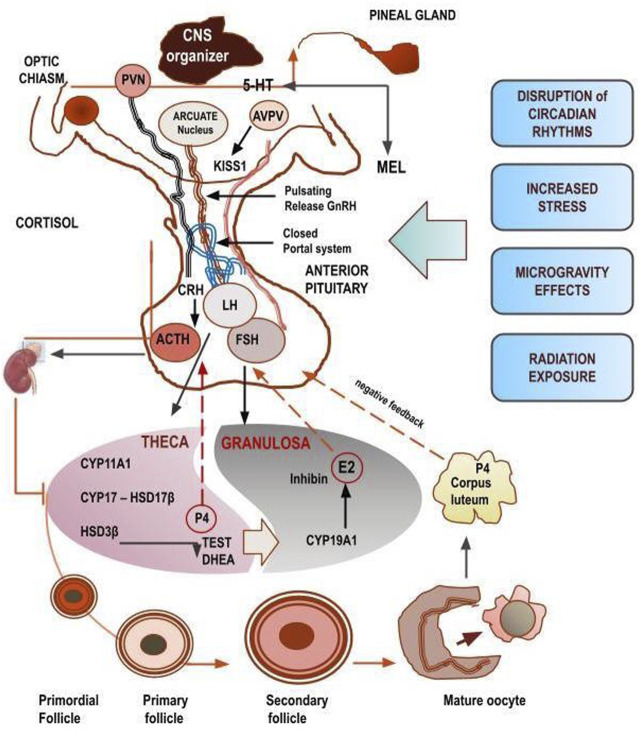
Regulatory tree of steroidogenesis distributed along a hierarchical axis. The diagram illustrates the main hubs that environmental space-related stressors can affect.

The female reproductive system consists of internal and external organs that are modulated by several nutritional, biophysical, endocrine and psychological factors, interacting across different and intertwined levels of organization. This apparatus is involved in regulating sex hormones (participating in several critical physiological functions), producing gametes and ensuring their subsequent maturation. This process is instrumental in the development of the zygote and subsequent fetus, ensuring the functional environment necessary for fetal growth and preparation for delivery. The female reproductive interval spans between menarche (the first occurrence of menstruation) and menopause (cessation of menses for 12 consecutive months): the interconnected endocrine network encompasses critical changes during the lifespan, also undergoing sophisticated modifications across time - the menstrual cycle. Noticeably, reproduction-associated processes entail several organs and tissues, not limited to sexual organs as other apparatus–including bone and cardiovascular systems–are tightly dependent from the endocrine ovarian control acting mostly through the release of the main steroid hormones 17β-estradiol (E2) and Progesterone (P4) (Figure 1).

Upstream of this process, ovarian steroidogenesis is extremely sensitive to the influence exerted by systemic neuroendocrine regulatory factors–including gonadotropin-releasing hormone (GnRH) and Kisspeptin, produced by the hypothalamic–pituitary–adrenal axis (HPA), as well as by paracrine cues, which include a plethora of signaling factors (Bone Morphogenetic Protein, Activin, Inhibin, Melatonin) (Zavala et al., 2020).

Kisspeptin encoded by the KiSS-1 gene plays a pivotal role in gametogenesis and endocrine regulation by modulating the HPA axis, inducing the release of GnRH and being inhibited, in a negative feedback cycle, by sex hormones such as testosterone and 17β-estradiol (Smith et al., 2005). A preliminary study performed on a rodent model of tail-suspension hindlimb unloading to simulate the microgravity condition has demonstrated that Kisspeptin significantly decreases after 30 days of exposure (Moustafa, 2021). Albeit the study was carried out on male rats, results showed that the simulated weightlessness was able to also downregulate pituitary expression levels of FSH and LH, ultimately impairing spermatogenesis, suggesting the possibility of an influence on female steroidogenesis as well.

GnRH is released in a pulsatile manner by the hypothalamus and acts on the anterior pituitary lobe (Adeno-Hypophysis), modulating the synthesis and subsequent secretion of follicle-stimulating hormone (FSH) and luteinizing hormone (LH) into the bloodstream. In the follicle, downstream of the Hypothalamic-Pituitary-Gonadal axis (HPG), FSH and LH activate the steroidogenic pathway by promoting in the Theca cells the synthesis of androgens that are further converted in estrogens in Granulosa cells. Steroidogenesis operates according to the two-compartment model, whose effectiveness depends primarily on the activity of Aromatase (Cyp19A1), the enzyme responsible for the aromatization of androgens into estrogens (Estrone and 17β-Estradiol) (Stocco, 2012). Specifically, FSH induces the upregulation of aromatase in Granulosa cells, while LH stimulates the production of androgens in theca cells.

Estrogens and Progesterone are released in a cyclic manner under the influence of LH/FSH and, in turn, they exert negative feedback onto the hypothalamus and anterior pituitary. A relevant exception is the positive feedback deployed by E2 during the ovulation, thus increasing the GnRH pulse frequency and stimulating the LH surge.

Additionally, female behavior during mating shows variable sexual susceptibility depending on endocrine inputs (Yin and Lin, 2023). Several hypothalamic areas, located especially in ventrolateral and ventromedial hypothalamus, display a specific distribution of estrogen receptors (ER) type 1, which are involved in controlling mating receptivity (Liu et al., 2022), according to a complex dynamic (Liu et al., 2024).

Overall, the menstrual dynamics reflect many underlying complex interactions, involving ovarian steroidogenesis, scheduled recruitment of follicles ending with the maturation of the oocyte, and contemporary changes in the endometrium, which are driven by central hormones (release by HPG axis), triggering many feedback loops encompassing molecular and biophysical factors. Noteworthy, the entire system is tightly synchronized with the circadian rhythm, alongside with both central and peripheral clock genes.

Spaceflight conditions likely may disrupt this delicate control, by acting through different and convergent mechanisms. Alteration of the sleep-wake rhythm and alteration of circadian rhythms impair the physiological release of melatonin and other several neurotransmitters. It is worth mentioning that several hypothalamic neurohormones (including serotonin, catecholamines, and oxytocin) are modified after exposure to microgravity (Blanc et al., 1998), (Kastello and Sothmann, 1999), (García-Ovejero et al., 2001), although the mechanisms responsible for these changes are not yet known. Circadian rhythm disruption is one of the main causes of fewer chances for reproductive success, given that some critical functions, such as body temperature, heart rate, and hormone secretion are tuned and regulated according to the chronobiological rhythm (Sciarra et al., 2020). Specifically, loss of rhythm in the suprachiasmatic nuclei–tightly modulated by the alternate cycle of appropriate dark/light periods - can affect the pulsatile sexual hormones release (Dickmeis et al., 2013).

Glucocorticoids produced by adrenal glands function according to a circadian rhythm and participate in the synchronization of the endogenous clocks. Stress conditions and disruption of circadian rhythms steadily increase cortisol levels and impair many functions, including the reproductive system (Domes et al., 2024). Noticeably, cortisol inhibits GnRH release upon the hypothalamus and influences gonadotropins synthesis and release on the pituitary (Vitellius et al., 2018). Consequently, glucocorticoids can cause alteration of ovarian cyclicity, by either inhibiting Kisspeptin neurons or stimulating the release of gonadotropin-inhibitory hormone (Michael et al., 1993). As expected, both salivary and blood/urine levels of cortisol show a significant increase during spaceflight missions (Zangheri et al., 2019), (Benjamin et al., 2016), and it is tempting to speculate that this effect can impair the reproductive function.

Physical exercise is a further confounding issue. On Earth, HPG axis activation is tightly dependent upon both the extent and intensity of exercise (Tremblay et al., 1985) and can even lead to amenorrhea/anovulation (Hakimi and Cameron, 2017). Nutritional deficits can worsen the HPG response further (Williams et al., 2015), eventually leading to a relevant disruption of reproductive hormones balance. Therefore, as women in space undergo a stressful program of physical exercise–required to counteract the negative impact of weightlessness on bone and muscle–it is possible that intense physical exercise in space could alter the HPG response, though there is no evidence of exercise in space leading to amenorrhea/anovulation to date.

Thus, it is conceivable that exposure to microgravity, as well to other harmful spaceflight conditions, can significantly interfere with proper reproductive function and steroidogenesis. Noticeably, modifications of reproductive capabilities can occur at multiple levels and do not involve only endocrine organs (ovaries, testis), but may happen at the hypothalamus or pituitary.

Due to the complexity of the system under scrutiny, as well as the different mechanisms of action triggered by spaceflight, investigating this intricate question is arduous and requires the use of several and appropriate experimental models.

## Models

Until now, limited research has focused on the effect of space travel on the reproductive system and its function, along with endocrine regulation of reproduction or prenatal development (Jain et al., 2023). A fundamental preliminary issue lies in establishing a proper model of investigation, a task essential for identifying the impact of specific stressors. Indeed, it is problematic to distinguish whether reproductive changes are due either to modified gravity or to radiation–the two most relevant threats–or should also be ascribed to other factors, such as noise, isolation, disrupted circadian rhythms, and stress (Monga and Gorwill, 1990). It is worth remembering that ovarian steroidogenesis undergoes modifications throughout a woman’s lifespan. Therefore, it is imperative to correlate experimental data to women’s age, and with the concomitant hormonal changes, especially those occurring during critical periods (perimenopausal and postmenopausal periods).

In addition, the short duration of spaceflights, and the small number of women who have participated in at least one space mission, limits the statistical validity of such studies. Moreover, it can be reliably assumed that short and long exposure to space conditions may have different (and eventually unexpected) effects on reproductive health. Indeed, in both male animals and astronauts, microgravity showed to influence the HPG axis response only during exposure for short periods. In rodents, plasma and tissue testosterone decreased after 2 weeks of spaceflight (Amann et al., 1985), (Grindeland et al., 1990). Similarly, testosterone decreased in astronauts after 12 days of weightlessness. In contrast, quite paradoxically, testosterone did not change during prolonged stay in space, while, after landing, it decreased up to 50% of basal values (Smith et al., 2012).

By no doubt, the simultaneous exposure to multiple environmental hazards complicates the assessment of the relative weight of each risk factor and their mutual synergy. It is worth mentioning that studies conducted during spaceflights have been performed in Low Earth Orbit (LEO), where radiation exposure is lower compared to that in outer space.

Therefore, various ground-based models involving either animals or *in vitro* cells are frequently used for investigating the bone and musculoskeletal systems (Emily, 1979); they also have been employed to assess the adaptive and pathological response to microgravity of the reproductive apparatus. Using these models offers several advantages, including practical feasibility, the possibility to control environmental factors in a more precise manner, as well as the possibility to conduct experiments repeatedly at minimal cost. However, these opportunities present a double-edged nature, as animals adapt rapidly to these stressing conditions, thereby dabbing the physiological stress response in short times (Ortiz et al., 1999). Therefore, ground-based models could be questionable as simulated microgravity models are bias-affected by considering gravity a force rather than a field.

Moreover, astronauts are vulnerable to both reduced and increased gravity exposures, and this could represent an additional source of uncertainty in identifying exactly the impact of weightlessness upon the reproductive system. In addition, different microgravity values are expected when different space environments are considered: on the surfaces of the Moon and Mars, and on the ISS, the intensity of the gravitational field varies and is lower compared to Earth, tending towards zero in the latter case, due to the free fall regime (Williams, 2015). It is therefore conceivable that organisms, in their macroscopic complexity, and cells, locally, would respond in a very different manner in those different circumstances. (Wolfe and Rummel, 1992).

## Female reproductive effects

### Animal data

The first studies focusing on mammalian reproduction were performed on unmanned satellites. A pool of male and female rats experienced 19 days onboard of the COSMOS-1129 biosatellite, in which the sexual encounters were allowed after removing a physical barrier. Once back on land, they guessed that two out of five females underwent pregnancy with resorption, while the remaining three rats showed signs of estrus disturbance, lack of ovulation and other problems related to fertilization (Serova and Denisova, 1982).

In a subsequent, similar study pregnant rats were sent during the COSMOS-1514 biosatellite mission for some days in space. On the re-entry, they showed reduced fetal weight and skeletal development when compared to ground controls (Serova et al., 1984). In addition, litter from flight mothers showed increased mortality during the first postnatal week. Two experimental investigations (study NIH. R1 and NIH. R2) focused on the pregnancy outcome of Sprague–Dawley rats that spent 2 weeks in space. After landing, animals were sacrificed, whereas the pups were assigned to on ground-raised dams. Spaceflight did not alter main pregnancy (maternal weight gain during pregnancy, duration of pregnancy, duration of parturition, litter size) and hormonal parameters (serum LH, FSH, and P4), when compared to either synchronous flight or unilateral hysterectomy vivarium controls, albeit pituitary LH content decreased (Burden et al., 1997). Some other interesting modifications were recorded in myometrium structure, given that the major gap junction protein (connexin 43) – required for enabling the electrical coupling of myometrial cells - was decreased in the flight group *postpartum* compared to controls (Burden et al., 1998). Tissue estrogen and progesterone receptor expression were both lower than that assessed in animals in ground controls. However, by examining in detail the experimental plan of the aforementioned studies we can detect many potential factors of bias. First animals had a laparotomy before (to make sure they had enough developing embryos) and post-flight, before being allowed to labor. This complicated procedure may have significantly altered the results, not to mention the stress the animals had to undergo.

Further studies performed during sequential missions (STS-131, 133, and 135/BSP) showed that female mice had reduced ovary weights than controls (Ronca et al., 2014). The ovaries were significantly smaller in flight (4.04 + 1.53 mg) compared to ground controls (5.52 + 0.89 mg), with fewer corpora lutea in the flight ovaries. Moreover, several growing follicles were atretic, indicating blocked estrous cycle. Similar results have been recorded during three subsequent missions (STS-131, STS-133, STS-135), confirming a serious impairment of the corpora lutea and the significant reduction of estrogen receptors in the uterus (Holets et al., 2015). Remarkably, expression of estrogen receptors ERα and ERβ were significantly lower (>20-fold) in the uteri of animals exposed to spaceflight. Similarly, progesterone receptor Pgr-AB expression was downregulated (3–4 fold) in flight ovaries. Reduced expression of estrogen receptors was further confirmed by the recorded downregulation of lactoferrin expression, as both ERα and ERβ are known positive regulators of lactoferrin (Teng et al., 2002).

Intriguingly, some precursors of the tryptophan-melatonin pathways (5-Hydroxy-Indoloacetic acid and 5-Hydroxy-Tryptamine) were found increased in mice during Cosmos1887-Bion eight and the Cosmos 2044-Bion nine spaceflight missions (Charles et al., 2015), (Holley et al., 1991). The overall results of the latter study indicate that animals experienced a significant stress as witnessed by adrenal hypertrophy and depletion of pineal melatonin stores, associated with an increased turnover of several intermediate metabolites of the serotoninergic pathway. According to the author’s conclusions, melatonin may have been released in large quantities due to spaceflight-related stress, resulting in pineal depletion of the neurohormones. In turn, increased release of melatonin could exert negative effects upon steroidogenesis, namely by inhibiting the hypothalamic release of GnRH, with a consequent alteration of FSH and LH levels (Basini and Grasselli, 2024).

Taken together, data provided by experimentation on mice suggest that the spaceflight environment may affect the reproductive system, by interfering with several hormone signaling. However, the reduction in estrogen availability could be an underlying mechanism explaining changes on wider physiological systems occurring during spaceflight (Tash et al., 2011). Moreover, changes in estrogen availability might likely influence sexual arousal and, ultimately, pregnancy potentialities.

Overall, the studies performed in the years 1965–2000 with animal models demonstrated potentially serious implications for human reproductive capability in microgravity conditions. Namely, investigations utilizing mammalian species concordantly reported significant alterations in morphology and development of both male and female germ cells (Keefe et al., 1981), (Plakhuta-Plakutina et al., 1976). These findings could have significant implications for embryogenesis, potentially leading to a significant number of abnormalities and/or aborted fetuses. The preliminary conclusions were rather pessimistic, stating, «The problems of embryogenesis or later child development may prove to be insurmountable in a zero—gravity environment due to the significant contribution of gravity to the normal development of bone and muscle. Planetary surfaces with higher or lower levels of gravity compared to Earth may also result in developmental abnormalities *in utero* or after birth» (Santy and Jennings, 1992). However, the modulation of endocrine pathways and the regulation of embryogenesis under gravity regimes distinct from Earth’s gravity or microgravity remain a largely unexplored area of research.

Intrinsic, methodological limits of experiments carried out in this period were unsurmountable and, as highlighted by many scientists, asked for a more appropriate investigation model in which the entire cycle of mammalian development could be studied in the space environment (Serova et al., 1982).

A highly used device for investigating microgravity effects on rodents is the Morey-Holton hindlimb suspension (HLS) model. This model–first described in the 1970s - involves suspending rats by the tail base to produce a 30° head-down position, a condition comparable to the human 6° head-down tilt utilized in bed-rest studies (Morey-Holton and Globus, 1985). This position results in a cephalic fluid shift and musculoskeletal unloading, which also occur during spaceflight. The intent of developing a hindlimb unloading model is to provide a ground-based system that simulates certain aspects of spaceflight in animals. The first model did not allow for ambulatory activity and provoked a significant stress response in the animals. To overcome this important limitation, HLS models continue to be modified in order to minimize stress and to allow either horizontal or head-down unloading to investigate the consequences of fluid shifts (Musacchia, 1985).

The HLS model was used to simulate the major physiological effects of hypo gravity on pregnancy. Exposure of rats to HLS during the early phases of pregnancy reduces implantation (Gharbi et al., 1996). Intriguingly, in this condition rats did not experience a significant distress, as documented by the lack of elevated plasma or adrenal corticosterone, suggesting that the impaired reproductive capabilities were due to microgravity effects only, rather than to secondary effects of stress (Kinoue, 1996).

In female Sprague-Dawley rats, the simulated weightlessness prolonged the diestrus and lengthened estrous cycles, suggesting that animals experienced hypoestrogenism (Tou et al., 2004). Remarkably, the length of these cycle’s phases resulted significantly modulated by diet, given that commercial chow showed to attenuate hypoestrogenism (likely by providing an exogenous source of estrogens). Simulated weightlessness is reputed to «activate» the HPA axis, as confirmed by increased urinary cortisol levels during the first experimental days, thus suggesting that the response to acute stress constitute a feasible mechanism explaining the disruption of estrous cycles. It is worth noting that in chow-fed rats, the increase in urinary cortisol resulted unaffected, thus suggesting that estrogens in chow-diet do not antagonize the stress response.

Hormonal data collected after spaceflight have provided controversial results, with reduced hypophysis content of LH, while serum LH concentrations resulted in normal range (Burden et al., 1997). Similarly, FSH in the pituitary showed a significant reduction whereas the plasma content steadily increases. Likely, a more realistic analysis would benefit from a sequential blood sampling as both LH and FSH display a pulsatile secretion, imposing to reconstruct a circadian profile for an appropriate assessment. Indeed, some reports showed that rats mated during spaceflight ovulated and cycled normally; however, in that study mating did not give rise to birth (Serova and Denisova, 1982). Autoptic examination performed post flight evidenced that fetuses could be resorbed, without clear proof of whether conception or implantation could have occurred in space. Therefore, there is still a lack of scientific evidence regarding conception and implantation mechanisms under stress conditions, such as microgravity, suggesting that this represents a significant issue that still requires many answers.

Studies performed to assess the consequences of spaceflight on pregnancy provided inconclusive results. Some spaceflight missions have included rat dams for different times between days 9–19 of the 22-day gestation period (Amann et al., 1985), (Ronca and Alberts, 2000), (Wong and DeSantis, 1997). Overall, these studies recorded minor changes in respect to control dams, besides a reduction in offspring weight gain, prolonged parturition, lower birth weights, and increased perinatal mortality were recorded in just one study (Jain et al., 2023). In this case, the environmental stress may have contributed to the increased natal mortality, a finding that persisted in the F2 generation, as evidenced by several publications (Herrenkohl and Gala, 1979).

Microgravity may also interfere with oocyte maturation throughout the ovarian folliculogenesis, although this issue has attracted scarce attention. Mammal females have a limited number of oocytes throughout their life span, and the count steadily decreases as age advances. Oocytes are actively nurtured during folliculogenesis, a complex process involving systemic endocrine and intra-ovarian control, displaying gonadotropin-independent and gonadotropin-dependent phases, regulated by hormones (LH, FSH, ACTH), intra-ovarian endocrine factors (melatonin, AMH, Activin, Inhibin), cytokines (growth differentiation factor-9 - GDF-9 – and bone-morphogenetic protein-15 - BMP-15), inositol, growth factors, calcium fluxes, gap-junction proteins and cytoskeleton components (Orisaka et al., 2021). This complex framework undergoes time-dependent changes according to a non-linear dynamic, which cannot be described by reductionist models since they fail to capture sophisticated phenomena like those following the removal of the gravitational constraint (Echenim et al., 2005).

Simulated weightlessness reproduced using rotating wall vessels showed that proliferation of granulosa cells obtained from porcine ovaries was severely inhibited, with consequent arrest of the cell cycle (Dai et al., 2021). This effect is associated with other abnormalities related to cell-to-cell communication, resulting in the disruption of intercellular communications. Deregulation among theca and granulosa cells therefore impair the folliculogenesis, as witnessed by the increased rate of atresia in ovaries flown in space (Holets et al., 2015).

In mice exposed to simulated weightlessness, the rate of oocyte maturation significantly dropped (from 8.94% *versus* 73.0% in controls), probably because of a disruption of the meiotic spindle organization (Wu et al., 2011). This study - specifically designed to minimize shear stress (a frequently overlooked factor that can lead to biased results for on ground-based models) - showed delayed maturation of oocytes harvested at the first meiotic division from immature mice exposed to weightlessness, albeit oocytes were properly stimulated with FSH, LH and 17β-estradiol for 16 h. In addition, since the intercellular communications (GCs transzonal projections and oocyte microvilli) were found severely disrupted in microgravity, it was hypothesized that these abnormalities may decrease oocyte quality due to the lack of morphogenetic factors (Cheng et al., 2023). Those findings have been vindicated by an investigation showing that the ultrastructural morphology of mitochondria, endoplasmic reticulum, and cortical granules of human oocytes exposed to simulated weightlessness are severely disrupted, probably because a rewiring of the cytoskeleton (Miglietta et al., 2023). Additionally, using a rotatory system device to reproduce simulated weightlessness, several ultrastructural abnormalities of the oocyte, reduced follicle survival and downregulation of GDF-9 expression were found (Zhang et al., 2016). It is worth mentioning that the study highlighted that follicles embedded in a three-dimensional microenvironment (alginate) survived significantly longer, showing a behavior superimposable to follicles growing on normal gravity. This finding suggests that the impairment of mechanotransduction–apparently « restored » by enabling cells to interact with a solid, surrounding environment–could be considered a primary causative factor in weightlessness-related effects on living organisms (Bizzarri et al., 2022). It is worth of mention that the Rotary Cell Culture System (RCCS) is a bioreactor designed to simulate microgravity conditions for cell culture. (Mitteregger et al., 1999). The system uses gentle fluid dynamics to maintain cells in a dynamic suspension, promoting spheroid formation while minimizing shear stress. However, this device has relevant limitations. These include potential for cell sedimentation at high speeds, requires a dynamic fluid suspension for cell culture (promoting hence some hydrodynamic forces), limited velocity ranges, and challenges with large cell aggregates and mass transfer (Talukdar et al., 2014).

The involvement of GDF-9 it is worth of interest given that–in association with IGF-1–it stimulates the replication of theca cells in small follicles (3–6 mm) from bovine ovaries, while depressing steroidogenesis (by reducing androstenedione and progesterone availability) (Spicer et al., 2008). Consequently, in theca cells from developing follicles, mRNA expression related to LH receptors and CYP11A1 was significantly downregulated by GDF-9, which is synthesized by granulosa cells. In addition, GDF-9 (associated with BMP-4) promotes granulosa cell proliferation and prevents premature differentiation of the granulosa cells during growth of follicles (Spicer et al., 2006). Reduced synthesis of GDF-9 in microgravity may therefore impair the cross talk between theca and granulosa cells, leading to disruption of both the steroidogenic pathway and the morpho-functional relationship inherent in the follicle’s two-compartment structure.

Until now, the effects of the space environment on the ovaries of non-pregnant animal females have not received the attention they deserve. The analysis of the ovaries of *postpartum* rats flown in space do not report significant changes regarding macroscopic parameters of ovarian function (weight, number of atretic or preovulatory follicles) (Ying et al., 1973).

Limited data are available regarding the HPA axis activity during spaceflight. A single study reported several disturbances and a clear reduction in serum GnRH, FSH, LH and testosterone in male rats exposed to weightlessness (Yin and Lin, 2023). Unfortunately, female rats were excluded from this experiment. These findings have been ascribed to an imbalance in cephalad-fluid shift occurring in microgravity, as the fluid redistribution probably induces pituitary deformation, as documented by MRI investigation performed during a spaceflight (Kramer et al., 2020).

### Female data

There is limited knowledge available on the systemic effects of spaceflight stressors, e.g. a wide range of diverse factors acting through different mechanisms - including altered gravity (micro-, hypo-as well as hyper-gravity), exposure to space radiation, confinement, disruption of chronobiological rhythms, changes in diet and physical activity. Overall, those leads can affect the HPG axis and significantly impair the functionality of the reproductive system. The limited available data related to women that experienced spaceflight represents a severe hurdle in assessing whether the space environment could compromise the fertility potential, albeit indirect information of female astronauts who flew during the Shuttle era evidence that the reproductive capabilities do not seem to be impaired by spaceflight.

Extreme states of stress can affect the menstrual cycle on Earth, and it is unknown if this would have the same impact on natural menstrual cycles in space. Data from female astronauts who flew during the Shuttle era suggest that pregnancy rates, including complication rates, are equivalent to ground-based, age-matched controls.

Indeed, after spaceflight some women were able to conceive (with or without the support of assisted reproductive technology), albeit several unsuccessful attempts have been recorded. Overall, data are scarce and do not allow for assessment of the real impact of microgravity and radiation exposure on the female reproductive capabilities. However, menstrual cycle irregularities have been reported in female flight attendants (Lauria et al., 2006), (Radowicka et al., 2013) - with an incidence ranging from 20% to 30%.

Considering that on board of the ISS the alternation of sunlight and darkness consists of ∼90-min intervals due to the orbital journey around the Earth, changes in the circadian rhythm interfere with GnRH signaling and can impair HPG control upon ovarian steroidogenesis. This unusual condition disrupts the sleep module (frequency/duration/quality), with consequent effects on the reproductive axis, as confirmed by studies performed on ground in which menstrual irregularities, as well as many other dysregulations (miscarriage, preeclampsia, preterm birth), are associated with sleep disturbances (Baker and Driver, 2007), (Caetano et al., 2021). Deregulation of circadian clock involves also specific genes at cellular levels, as exposure to microgravity modifies the expression of several clock genes (Bmal1 and Rev-erba among others), thus highlighting the existence of an integrated signaling network between the gravitational field and the circadian gene regulation (Ranieri et al., 2015). Clock genes are expressed in many tissues and organs, including ovaries where they participate in the regulation of key steroidogenic genes, including Cyp19A1 (Fujita et al., 2020).

Furthermore, female astronauts during space missions use contraceptives to suppress menstruation, thus preventing in assessing ovarian function and the consequent effects upon the endometrium. The combined oral contraceptive (COC) pill is one medication usually used by female astronauts, though there is no official requirement for using contraceptives. Although we have extensive knowledge about the physiological effects of the COC on Earth, there has been no extensive investigation regarding benefits/disadvantages in space. Use of COC would not only avoid unwarranted pregnancy but are instrumental to prevent menstrual problems that could be unmanageable during spaceflight missions (Grandi et al., 2012). Young women frequently suffer from menstrual pain and dysmenorrhea, affecting more than 50% of women (Proctor et al., 2001). The combined oral contraceptive pill, norgestrel and ethinyl estradiol, are recommended in the dose of 30–35 mcg COCs (taken continuously) to provide better suppression of the ovary (less chance for cysts and breakthrough bleeding, depending upon OC formulation) (Murad, 2008).

Noticeably, we should keep caution with contraceptives that suppress ovarian function for extended periods (like contraceptives containing medroxyprogesterone acetate, Depo-Provera or norethisterone enanthate), given that they have been shown to adversely affect women’s bone density and cardiovascular risk on Earth (Nappi et al., 2012). Therefore, as continuous use of COCs may inhibit ovulation, and reduce estrogens, it is tempting to speculate if COCs treatment for very lingering periods could be a suitable strategy for long-duration missions.

However, as assumption of COC inhibits ovulation, assessment of steroidogenesis would likely be biased by the concomitant hormonal treatment.

Since spaceflight is associated with reduced hemoglobin levels, menstrual irregularities may likely predispose astronauts to developing anemia (Jennings and Baker, 2000). However, while utilization of COCs to suppress menses may decrease the risk of inflight anemia, use of COCs have now been found to increase the risk of venous thrombosis, particularly amongst women with a genetic predisposition (Sehovic and Smith, 2010), (Lo et al., 2024). This problem deserves special attention: given that the first episode of venous thromboembolism (VTE) occurred in space in 2019, it is critical to investigate those factors which may further increase the risk associated with coagulation. Indeed, a recent study focused on venous thromboembolism events in female astronauts, showing an association between oral contraceptive use and serum albumin, among other factors, which potentially increase the risk of venous thromboembolism in astronauts (Zwart et al., 1985). Oxytocin can influence coagulation, potentially affecting venous thromboembolism (VTE) risk, also by increasing the probability of clot formation (Golparvar et al., 2014).

It is worth mentioning that oxytocin–a hormone involved in the modulation of HPA axis response to chemical and environmental stresses (Suh et al., 1986) – is reduced during spaceflight (Uno et al., 1997) and after COC assumption (Salonia et al., 2005). The two factors can likely synergize in reducing oxytocin in women involved in spaceflight missions and thus can further impair the adaptive response to stress and, consequently, reproductive fitness. Furthermore, considering that some contraceptives (like injectable depot medroxyprogesterone acetate) can reduce bone mass and impair calcium metabolism (Ampatzis et al., 2022), they are no longer prescribed to female astronauts. The impact of COC upon bone is currently an open issue, complicated by several concurring characteristics (posology, concentration of estrogen, and age of user) (Prior et al., 2001). To our knowledge, only a single study has examined the impact of spaceflight on bone structural qualities in female astronauts, showing that after prolonged space missions (49–215 days), male and female crew members displayed no relevant differences in bone density decrease, accounting −1.5% in Bone Mineral Density per months, regardless of sex and exercise modality (Smith et al., 2014).

Historically, fertility outcomes following space travel have been relatively poor. Success rates for assisted reproductive technology (ART) in female astronauts have been markedly low, when compared to age-matched controls (Ampatzis et al., 2022), albeit such results have been critically revised (and mitigated) by the same authors in a more recent update, in which it is concluded that « Long-duration exploration spaceflight will introduce new challenges for maintenance of gynecological and reproductive health. The impact of the space environment outside of LEO on gynecological concerns remains unknown, with factors such as increased radiation exposure adding complexity and potential risk» (Steller et al., 2020).

These findings reinforce the need for a specific women’s health database reporting clinical and biochemical data related to spaceflight.

Difficulties in obtaining evidence from women flying in space have prompted the use of models based on simulated weightlessness, including the six-head-down-tilt bed-rest model.

This approach has been employed in several studies, but the results gathered up to now are scarce and biased by the lack of a clear methodological rationale (Tou et al., 2002). The first study using the bed-rest model was unable to evidence any significant change, evidencing only minor modifications related to the secretion of Progesterone during the luteal phase (Sandler and Winters, 1978). The study, performed with an on-ground model, is unfortunately affected by an evident bias, as the observational period was restricted to only 17 days. This fails to account for the entire menstrual cycle and thus makes it difficult to provide a more complete interpretation of physiological changes experienced by women during this timeframe.

A further study reported that women exposed to weightlessness according to the bed-rest model showed luteal phase deficiency, interpreted as caused by HPA axis dysfunction (Rock and Fortney, 1984).

Another model that is gaining momentum is the «dry immersion» approach (Navasiolava et al., 2011), (Watenpaugh, 2016). However, as already noticed for other experimental models, the primary focus of these studies was to assess the musculoskeletal, nervous, and cardiovascular systems, and information about reproductive function was practically incidental (Robin et al., 2022), (Linossier et al., 2022).

Some recent reports based on different study designs have investigated the impact of simulated weightlessness performed by dry immersion. The experimental plan greatly differs among these studies, as the dry immersion period ranged from three to 5 days, occurring either between day 7 and 10 or between day 10 and 15 of subject’s menstrual cycles (Gorbacheva et al., 2023), (Tomilovskaya et al., 2019). Noticeably, the paper from Gorbacheva et al. observed increased Inhibin-B (that selectively suppresses the secretion of pituitary FSH) (Luisi et al., 2005) levels and decreased LH and P4 at day 9 of the menstrual cycle after the immersion. Unfortunately, Estrogen levels were not evaluated by the study. Remarkably, the ovarian volume after prolonged immersion decreased significantly (−22%), while an increase in dominant follicle diameter was reported.

## Effects of simulated microgravity on ovarian function in space

Ovaries play a central role in the control of female fertility: namely, the delicate balance between ovarian steroid hormones reflects the underlying driving influence of many systems and environmental factors–psycho-physical stresses, nutritional changes, drugs and immune stimuli - mostly acting through the apical endocrine control exercised by the HPG axis (Toufexis et al., 2014). Moreover, intrinsic and systemic endocrine and paracrine cues orchestrate menstruation in a cyclical manner, according to a complex mechanism involving several hormones, like Insulin (Ins), Luteinizing hormone (LH), Gonadotropin Releasing Hormone (GnRH), Follicle Stimulating Hormone (FSH), which dynamically interact with ovarian signaling pathways, involving Progesterone (P4) and 17β-Estradiol among others. The menstrual cycle travels through distinctive phases - proliferation, decidualization, inflammation, apoptosis, hemostasis, vasoconstriction, hypoxia, repair, and regeneration–that are instrumental in shaping the endometrium according to the aims required. This is why the ovarian cycle runs in parallel to the menstrual cycle, involving endocrine and structural changes for facilitating the maturation of a single oocyte. Once the oocyte has been released, corpus luteum emerges from the follicle and secrete P4 to support endometrial growth and maintenance of an eventual pregnancy. In the absence of fertilization, estrogens and P4 downfall and menstruation occurs.

**FIGURE 2:**
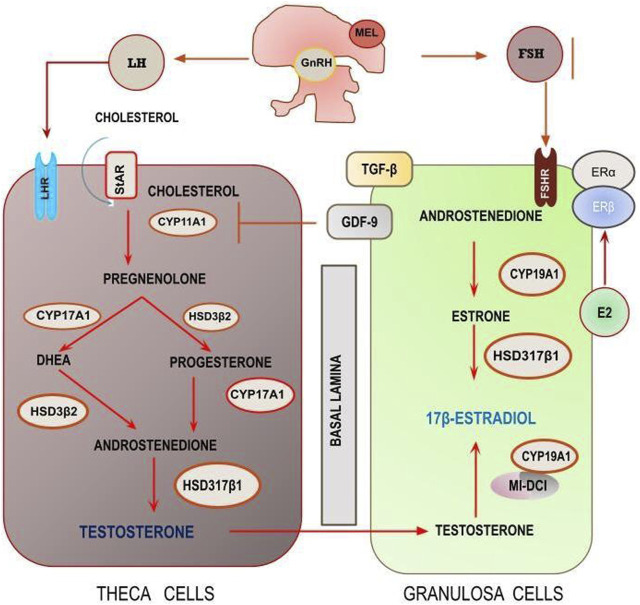
The steroidogenic pathway according to the two-compartment model. Theca cells synthetize androgens that, subsequently, are converted into estrogens. The entire process is under the regulatory control of both systemic (mediated by the activation of HPG) and local, chemical and biophysical factors.

Ovarian steroidogenesis is the main source of sex steroid hormones, produced through the interacting cooperativity between two distinct compartments: theca (TC) and granulosa cells (GC). Steroidogenesis begins with cholesterol as substrate for a number of subsequent transformations, catalyzed by specific enzymes and constrained by several factors belonging to both the internal and the external milieu, including hormonal mediators released by the HPG and the HPA (Figure 2). According to the two-cell two-gonadotropin theory (Armstrong et al., 1979), stimulation exerted by LH promotes the biosynthesis of androgens (DHEA, Androstenedione, and Testosterone) from cholesterol in Theca cells. Androgens then shuttle to GCs where, under the enzymatic activity of aromatase (CYP19A1), they are transformed into Estrogens (Estrone and 17β-Estradiol).

FSH is required to increase aromatase synthesis and activity. Estrogens produced by the ovary are needed to support the reproductive apparatus, but their role is not limited to the fertility field, as they are involved in maintaining cardiovascular efficiency, bone homeostasis, metabolism and immune functions (Davis et al., 2015). Particularly, available data suggest that weightlessness could impair estrogen synthesis and, for this reason, it is clear that estrogen deficiency may be a contributing factor to the worsening of the decrease in bone mineral density recorded during spaceflight (Stavnichuk et al., 2020).

Albeit no data (except from some anecdotal cases) (Kikina et al., 2024) are available about the steroidogenesis occurring in women ovaries during spaceflight, indirect data have shown that estrogen receptors–in different tissues - are modulated by the microgravity regimen (Mathyk BA. et al., 2024). This investigation evidenced significant changes in insulin as well estrogen signaling in murine and human cells exposed to microgravity. Noticeably, estrogen (including the CYP17A1 gene) and insulin-related genes were upregulated post-flight. An indirect confirmation of the disruption of estrogen signaling has been provided by an extensive study performed on rodents flown in space, in which transcriptomic analysis was carried out on OSD-511 dataset from NASA’s Open Science Data (Arnold et al., 2024). This study was able to evidence that microgravity severely downregulates Greb-1 - a gene regulated by estrogens and regulating cytoplasmic glycosyl-transferase that post-translationally stabilizes Erα (Shin et al., 2021).

These findings are intriguing, given that a correlation has been already suggested between dysregulated insulin signaling and ovarian steroidogenic function, namely in Polycystic Ovary Syndrome (PCOS) (Fedeli et al., 2023).

Polycystic ovary syndrome is the most common endocrine disease in women of reproductive age, and the principal cause of infertility (Yu et al., 2023). PCOS recognizes four distinct phenotypes, although the most frequent characteristics include hyperandrogenism with ovarian dysfunction, chronic oligo-anovulation, and/or polycystic morphology of the ovary. Noticeably, evidence shows that the reproductive function is severely impaired in ovaries from PCOS women, given that androgens (and related enzymes) increase, altogether with AMH (anti-Müllerian hormone), while estrogens and CYP19A1(aromatase) are significantly downregulated. As a consequence, granulosa cells are unable to efficiently transform the intra-ovarian androgen excess into estrogens *via* aromatase, thus leading to the arrest in follicular growth and anovulation (Fedeli et al., 2024). Given that aromatase function is highly sensitive to a number of environmental and psychological stresses, it is worth remembering that by this way the HPA axis can transduce negative inputs to the ovarian function (Fedeli et al., 2023), although a direct microgravity effect cannot be discarded, as exposure of granulosa cells in both real and simulated weightlessness showed a significant downregulation of CYP19A1.

Alteration of estrogen receptors were most prominent in the liver, where it resulted associated with a concomitant hepatic insulin resistance and steatosis. Hepatic steatosis can be ascribed to insulin resistance, which can worsen the transduction of insulin (Fabbrini and Magkos, 2015). Indeed, in the mentioned report it was shown that lipids accumulated into the liver, thus reinforcing the pathogenetic hypothesis. Even older studies showed higher plasma glucose and insulin levels after the oral glucose tolerance test in astronauts during short-lived spaceflight mission (Macho et al., 2003).

However, as estrogen receptors knockout is associated with the emergence of insulin resistance (Allard et al., 2019), it is tempting to speculate that estrogen deficiency may play a causative primary role in inducing insulin resistance. In any case, both insulin resistance and estrogen deficiency can impair the mitochondrial function. Interestingly, mitochondria activity is critically impaired during spaceflight (Tocci et al., 2024). Furthermore, Insulin resistance is associated with circadian rhythm changes and was recorded in both astronauts and animals flying in space (Aydogan Mathyk et al., 2021), (Hughson et al., 2016). Insulin signaling regulation is sensitive to many stressors, and its dysregulation affects folliculogenesis homeostasis. Impaired insulin transduction is recognized as a major factor in female infertility, as it can disrupt menstrual cyclicity and also lead to complications in pregnancy, leading to gestational diabetes (Lei et al., 2024).

The integrity of this complex system is mandatory not only to support a pregnancy but providing a balanced input of estrogens and progesterone, it ensures other relevant physiological tasks, belonging to the cardio-vascular and musculoskeletal system (den Ruijter and Kararigas, 2022), (Chidi-Ogbolu and Baar, 2019). Indeed, the most relevant homeostatic processes occurring within mammals–especially in females - are intensely influenced by gonadal hormones and this explains why fitness of the reproductive system is often considered a marker of overall health (Rajkovic and Pangas, 2017). Noticeably, steroid hormones play an indispensable support in modulating several physiological functions, including bone and cardiovascular homeostasis that are specifically affected by weightlessness. Unfortunately, research is still limited regarding the impact that microgravity could have on reproductive capabilities and its consequences upon other physiological functions (Mishra and Luderer, 2019).

Recently, NASA’s Rodent Research Hardware System (Rodent Research Habitat System) has been built to be used as a research platform aboard the ISS for long-duration rodent experiments in space. The platform hosted the Rodent Research-1, flown on the SpaceX-4 CRS-4 Dragon cargo spacecraft. This validation mission was the first to transport animals aboard the ISS in an unmanned vehicle (Hong et al., 2021). The experiment, lasting 37 days in microgravity, was the longest space rodent study conducted by NASA. Estrogen and progesterone - analyzed in the ovarian tissue and in serum - did not show significant modification among the different experimental arms. However, mice in flight had more animals in metestrus and diestrus, two stages in which the ovary usually displays reduced steroidogenic activity; whereas in control groups (vivarium and baseline control groups), all animals were found in proestrus and estrus, two stages associated with increased ovarian steroidogenesis due to the concomitant LH surge. It should be noted that the rodent estrous cycle lasts 4–5 days and it is divided into the aforementioned four phases, each lasting 1–2 days (Nowak et al., 2018). The overall analysis performed to investigate the gene expression pattern was unfortunately biased by the inclusion of stromal tissue and smaller follicles, which predominate over the selected follicles that become highly steroidogenic during the maturation process. Thereby, as stated by the authors, «when whole ovarian RNA is used, it was not surprising to see no major differences in ovarian gene expression across the treatment groups or even during different stages of the cycle».

The coordinated steroidogenic activity occurring between theca and granulosa cells is the ultimate process regulating reproduction as well as the release of relevant hormones, including estrogens and progesterone. TCs produce androgens then converted into estrogens in the GCs. Aromatase (encoded by CYP19A1) is the key enzyme in estrogen production by GCs.

Theca and granulosa cells capabilities and morphology in microgravity have not been specifically addressed, with the exception of the Ovospace experiment, flown in space during the 2022 Minerva mission, and the Orion experiment flown in 2024 as part of the AX-3 human mission by Axiom. The investigation focused on theca and granulosa cells growing into a minilab for 9 days (including the docking period). Preliminary results (Roiati et al., 2023), obtained from both in space experiments and in RPM-simulated microgravity, showed that steroidogenic enzymes belonging to GCs undergo a dramatic downregulation. Aromatase activity was almost completely suppressed, whereas CYP19A1 methylation (assessed either at CpG and non-CpG moieties) significantly increased. Overall, these findings indicate that aromatase activity–and consequently estrogen release–is severely downregulated, thus impairing the ovarian steroidogenic competency.

Noticeably, weightlessness seems to suppress aromatase activity, thus leading to a hypo-estrogenic condition. Reduction in estrogen synthesis not only impair ovulation and menstrual cycle, but also can likely affect bone and cardiovascular homeostasis. In this way, ovarian insufficiency can affect the functioning of many other systems, ultimately compromising health and performances of astronauts. Namely, evidence from three space shuttle missions (STS-131, STS-133, and STS-135) demonstrated that ovarian function is severely impaired in female animals. Ovarian folliculogenesis and luteal function were disrupted, as well as the uterine structure, likely because of dysregulated endocrine support (Ronca et al., 2014), (Holets et al., 2015). These findings derived from the spaceflight experiments raise questions as to whether pregnancy can be sustained in a reduced gravitational field (they want it in the discussion section).

The ovarian endocrine profile - so far underrated–deserves a proper assessment, by planning a well-designed experimental strategy. It is worthy of interest that the space-related deregulation involving ovarian steroidogenesis looks like the picture observed in the PCOS (Fedeli et al., 2024). These hints suggest that–as for PCOS–specific pharmaceutical countermeasures support could be envisaged to mitigate weightlessness-induced steroidogenic alteration (they want it in the discussion section).

## Conclusion

The primary challenge with any voyage or mission into space or to other planets is undoubtedly survival. The moment we leave our planet, we find ourselves in hostile territory: space is a dangerous field for human beings. At the very beginning of the Apollo program (1967), NASA operated without the concept of “permissible doses” of hazards–primarily focusing upon radiation–with the aim to prioritize success of the mission. This is no longer the case, as we now have tools and technologies to make space travel safer. Yet, several concerns arise as we are planning for a return to the Moon (Artemis program), and eventually to travel to Mars (Bizzarri et al., 2023). Protecting human reproduction capabilities belongs to these worries.

There is a severe paucity of data: preliminary results gathered during the last 50 years–in both animal and human studies - reveal that the space environment may have a significant impact on the reproductive cycle. Therefore, they ask for a confirmation. Indeed, much is required in order to acquire a full understanding of endocrine and functional changes occurring during microgravity exposure. Moreover, the joint effect of radiation and weightlessness deserve to be thoroughly investigated to recognize the respective contribution of each one as well as the eventual synergies. This implies that we have to design specific models–on ground as well as in flight–to perform extensive studies. Noticeably, how different gravitational regimens (like Moon and Mars where gravity account for 0.16 and 0.38 g, respectively) can affect reproduction needs to be specifically addressed and compared to results obtained on board of the ISS (in which the free-fall condition with less than 0.01 g is actually experienced by crew members). In this context, advancing technological efforts to establish facilities dedicated to long-term animal studies in orbit or on the Moon could significantly enhance our understanding of the effects of space adaptation on mammalian reproductive physiology.

Changes in gene expression, proteomic pattern, metabolomic analyses, as well as miRNAs release are required to fully attain an understandable picture of the whole (Corti et al., 2024). Moreover, studies involving both animals and 3D-cell models would require longer exposure either in space or in simulated conditions. In addition, novel modeling would benefit from the collection of biological samples in-flight to avoid the confounding effects due to several physical stresses related to the reentry. In particular, investigation should explore to what extent gravity-related changes can be considered an «acute transition state», anticipating an eventual «steady-state» that still has to be unveiled. Furthermore, it is still a matter of inquiry if the observed dysregulation in steroidogenic function is reversible and can finally allow recovery of normal reproductive function after prolonged exposure to microgravity.

Consequently, research into the reproductive function in space can provide useful important data regarding the properties of the reproductive process by highlighting critical targets and pathways, which are specifically affected by microgravity conditions. Furthermore, this field of research can potentially open new perspectives of investigation, including those aimed at improving fertilization assisted technologies, stem and germ cells management, oocyte maturation and detoxification from toxicant harmful for reproduction.
