# Correction: Microgravity impairs endocrine signaling and reproductive health of women. A narrative review

**DOI:** 10.3389/fphys.2025.1734194

**Published:** 2025-11-24

**Authors:** Michela Cutigni, Giorgia Cucina, Emanuele Galante, Matteo Cerri, Mariano Bizzarri

**Affiliations:** 1 Department of Experimental Medicine, Space Biomedicine Laboratory, University Sapienza, Rome, Italy; 2 Department of Biomedical and NeuroMotor Sciences, University of Bologna, Bologna, Italy

**Keywords:** weightlessness, steroidogenesis, aromatase, female fertility, ovarian function

An incorrect number was provided for **ASI Grant** “Ovospace”. The correct number is DC-DSR-UVS-2022-2-HH.0.

The original article has been updated.

